# Retracted: Elucidation of Genotypic Variability, Character Association, and Genetic Diversity for Stem Anatomy of Twelve Tossa Jute (Corchorus olitorius L.) Genotypes

**DOI:** 10.1155/2021/1301534

**Published:** 2021-02-19

**Authors:** BioMed Research International

*BioMed Research International* has retracted the article titled “Elucidation of Genotypic Variability, Character Association, and Genetic Diversity for Stem Anatomy of Twelve Tossa Jute (Corchorus olitorius L.) Genotypes” [1], due to significant textual overlap with the author's previously published article [2]:

Additionally, many of the figures and tables appear to overlap in these two articles:

Table 1 [1] overlaps with Table 1 [2]

Figure 1 [1] overlaps with Figure 1 [2]

Figure 2(b) [1] overlaps with Figure 2 [2]

Figure 3 [1] overlaps with Figure 3 [2]

Figure 2(a) [1] overlaps with Table 6 [2]

Table 2b [1] overlaps with Table 2 [2]

Figure 4 [1] overlaps with Figure 4 [2]

Table 4 [1] overlaps with Table 3 [2]

Table 7 [1] overlaps with Table 9 [2]

The authors disagree with this retraction.
